# A novel catheter with retractable stent that can prevent aortic insufficiency during left ventricular assist

**DOI:** 10.1371/journal.pone.0194658

**Published:** 2018-04-02

**Authors:** Jing Lin, Zhen Qin, Hong Qian, Yajiao Li, Nanfu Luo, Lei Du

**Affiliations:** 1 Department of Anesthesiology and Translational Neuroscience Center, West China Hospital, Sichuan University, Chengdu, China; 2 Department of Cardiovascular Surgery, West China Hospital, Sichuan University, Chengdu, China; 3 Department of Cardiology, West China Hospital, Sichuan University, Chengdu, China; University of Bern, University Hospital Bern, SWITZERLAND

## Abstract

Left ventricle (LV) assist, which refers to the use of a mini-pump or catheter implanted across the aortic valve connected to the pump, can promote myocardial recovery after left ventricle failure. However, conventional LV assist catheters compress the aortic valve, which can induce aortic insufficiency. Here we describe a novel LV assist catheter containing a retractable stent at its distal end that may prevent such insufficiency. The device was tested in six goats in which the coronary artery was ligated to induce acute LV failure, and then an LV assist was installed with a novel catheter in the left ventricle via the left subclavian artery. Inserting the catheter into the left ventricle caused mild to moderate aortic insufficiency. Releasing the stent maintained the catheter in the center of the three valve leaflets, which resolved the aortic insufficiency and, within a few minutes, led to significantly lower left ventricle end diastolic pressure (9.0±3.0 mmHg) than without stent release (17.6±5.0 mmHg, p = 0.012) as well as significantly higher left ventricle d*P*/dt_max_ (614±299 mmHg/s) than without stent release (343±245 mmHg/s, p = 0.03). Our results indicate that this novel drainage catheter with retractable stent can effectively prevent aortic insufficiency by maintaining the catheter in the center of the aortic valve leaflets, thereby lowering left ventricular end diastolic pressure and improving systolic function.

## Introduction

Temporary mechanical circulatory support is a well-established procedure to restore sufficient myocardial function in patients with cardiac failure, or to provide a bridge to cardiac transplantation. For patients with severe left heart failure, left ventricle (LV) assist is commonly used to increase myocardial perfusion and reduce oxygen consumption in the left side. LV assist pumps blood from the left side of the heart into the arterial system. This reduces preload of the LV. One approach to LV assist is transeptal placement of a cannula into the left atrium, but this fails to drain the LV sufficiently and leaves a residual atrial defect [1–2]. Another approach is to insert a catheter or small axial flow pump such as the Impella (Abiomed, Danvers, MA, USA) into the LV across the aortic valve through a peripheral artery [3,4]. Unfortunately, this approach is associated with development of native aortic insufficiency (AI)[5–7], such that a portion of pumped blood flows back into the LV. This increases left ventricle end diastolic pressure (LVEDP) and delays left heart perfusion, prolonging the recovery process and potentially making it impossible to wean the patient off LV support [8,9]. Long-term LV assist leads, in turn, to pathological structural changes in the aortic valves [6,10].

We hypothesized that it may be possible to reduce AI by maintaining the catheter in the middle of the three aortic leaflets to prevent the catheter from compressing the aortic valve. To test this hypothesis, we developed a retractable stent located on the distal end of an LV drainage catheter, such that deployment of the stent would help keep the tube centrally positioned in the valve leaflets. We validated this novel LV component in a goat model of LV failure.

## Methods

### Animals and anesthesia

This study was performed on six adult goats weighting 30–40 kg. Anesthesia was induced with 4 mg/kg propofol (2% Disoprivan, AstraZeneca, Germany) and 0.3 mcg/kg sulfentanil (Yichang Humanwell, China). Goats were ventilated (Datex-Ohmeda Excel 210, Soma Technology, Cheshire, CT, USA) at a fraction of inspired oxygen of 0.4 and respiratory frequency of 18 per minute. Tidal volume was adjusted to maintain arterial partial pressures of CO_2_ at 35–45 mmHg and O_2_ above 100 mmHg during the experiment. Anesthesia was maintained using 1–3% isoflurane inhalation as well as continuous infusions of propofol (60–120 mcg/kg/min), vecuronim bromide (0.2 mg/kg/h) and sufentanyl (0.03 mcg/kg/min). Body temperature was maintained at 36–37 °C throughout the experiment. After femoral vein cannulation, Ringer’s lactate solution was administered at 10–30 ml per kg per hour. At the end of the experiment, animals were euthanized with an overdose of sodium pentobarbital.

The animals used in this study were provided by West China Center of Medical Sciences, Sichuan University. Its location is No. 1, Gao Peng Avenue, Wuhou District, Chengdu, Sichuan. These animals were bred for research purpose. This study was approved by the Institutional Animal Care and Use Committee of Sichuan University(No.2015061A). The procedures were in compliance with the US National Institutes of Health Guide for the Care and Use of Laboratory Animals (NIH Publication No. 85–23, revised in 1996).

### Catheter design

**Fig 1:**
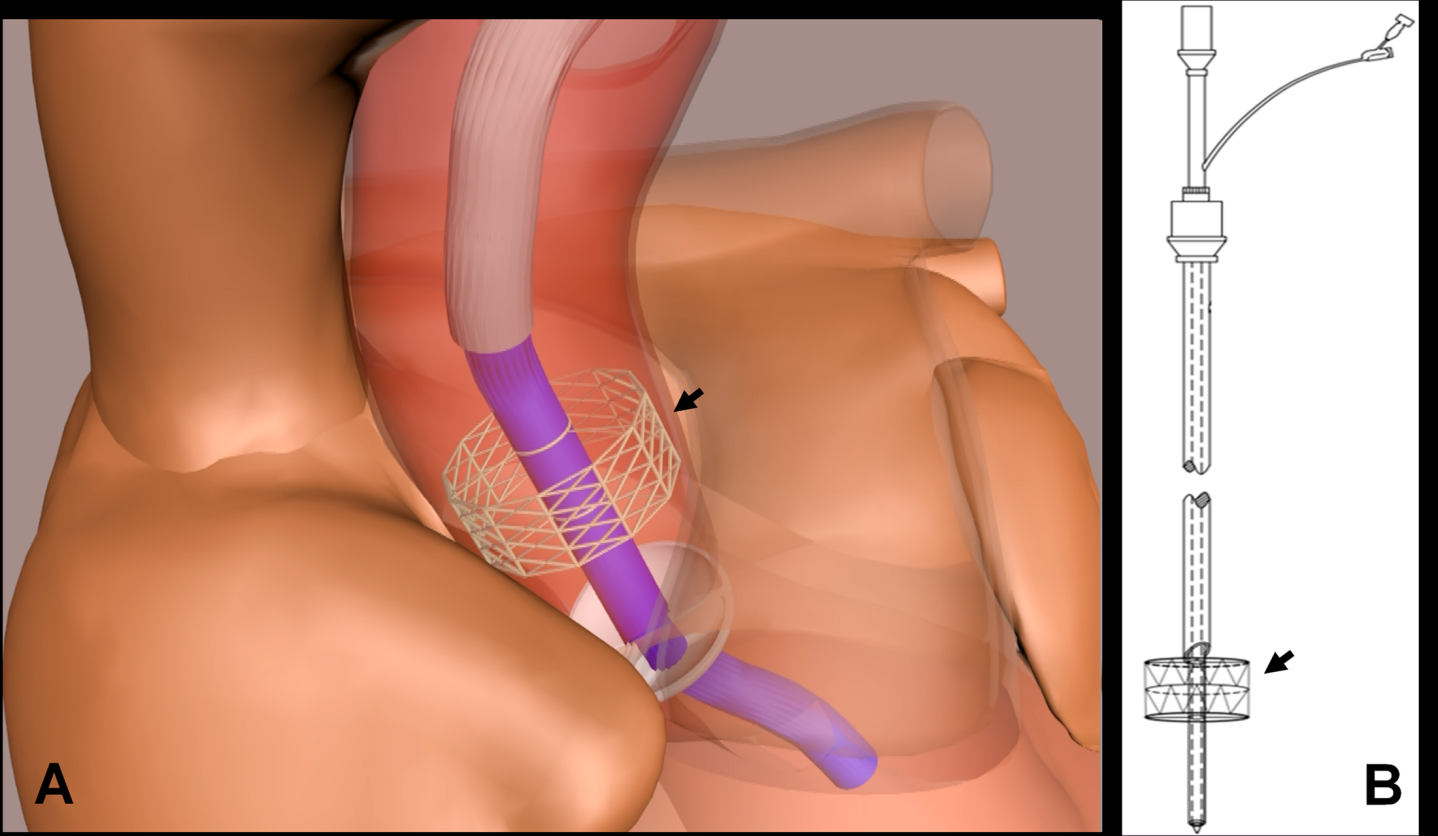
Schematic diagram of a novel drainage catheter. **A.** A stent (arrow) fixed at the distal end of the catheter for left ventricle venting. **B.** Release of a retractable vascular stent (arrow) at the distal end of the catheter props up the proximal aorta and maintains the catheter in the center of the aortic valve leaflets.

The LV drainage catheter (internal diameter, 4.7 mm; length, 60 cm) was designed to contain an outer tube, inner tube, introducer and stainless steel guide wire. The novel component of this catheter is a retractable stent located on the distal part of the inner tube, which allows the inner tube to remain centered within the three valve leaflets when the stent is released (Fig 1A and 1B).

### Induction of left heart failure and LV assist

Left cardiac dysfunction was established in goats by inducing myocardial ischemia as described [11]. Briefly, a mid-sternal incision was made to expose the heart, and the left anterior descending coronary artery was ligated and adjusted to induce cardiac dysfunction. The procedure was modified as needed because of anatomical variations in individual animals. Heart failure was confirmed when segmental motion of the LV was obviously abnormal by echocardiography (Vivid E9, GE Healthcare, Horten, Norway), and when the ejection fraction was less than 35% during sinus rhythm.

Systemic heparinization was carried out by administering a bolus of heparin (300 U per kg) intravenously in order to achieve an activated clotting time longer than 420 sec. The left subclavian artery was exposed, and a guide wire was inserted into the LV through the aortic valve. The novel catheter was inserted into the LV under wire guidance, then both the introducer and guide wire were removed, leaving the outer and inner tubes inside the blood vessel. Pulling back the outer tube allowed release of the retractable stent, which propped up the wall of the ascending aorta and thereby maintained the inner tube in the center of the ascending aorta (Fig 1B, S1 Video). All operations were performed under echocardiographic guidance.

To set up the LV assist, another femoral artery catheter (Edwards Lifesciences, Irvine, CA, USA) was inserted into the right femoral artery. A centrifugal pump (Medtronic, Minneapolis, MN, USA) was primed through injection with succinylated gelatin (Gelofusine^®^, B. Braun, Shenyang, China) and connected to the inner tube of the LV catheter and the femoral artery catheter. This LV assist withdrew blood from the LV and delivered it to the femoral artery. LV assist was initiated after induction of left heart failure and adjusted to maintain mean arterial pressure at 60–70 mmHg. At this point, rotational speed was fixed and it remained constant throughout the entire experiment, with and without stent release.

All animals underwent the same intervention involving induction of heart failure via ligation of the descending coronary artery, followed by insertion of the novel drainage catheter without and with stent release.

### Measurement of hemodynamics and aortic insufficiency

Arterial catheters (BD Bioscience, New Jersey, USA) were placed into the right femoral artery to record systemic blood pressure. A catheter tipped with a 5F micromanometer (Yixinda, Shenzhen, China) was inserted into the LV through the apex to measure its pressure. Pressures in the femoral artery and LV were continuously recorded using a multi-channel system (BL-420S, Taimeng Technology, Chengdu, China). Heart rate was measured by standard electrocardiography. Peak rates of change of LV pressure (d*P*/dt_max_) were extracted off-line from the LV pressure trace, which was recorded every minute for 5 min before induction of heart failure (baseline), after induction of heart failure, and after LV assist initiation with and without stent release.

Aortic valve regurgitation was evaluated by echocardiography before and after LV failure as well as during the use of LV assist with and without stent release. AI was accessed in both the long- and short-axis views. After each intervention, data were recorded after hemodynamics had remained stable for 10 min. Measurements were performed by two researchers, and mean values were used in statistical analysis.

### Statistical methods

All data were presented as the mean ± standard deviation of the values from all six animals. One-way ANOVA and paired Student’s *t* tests were used to assess the significance of differences among the four experimental conditions of baseline (before induction of left heart failure), immediately after heart failure, LV assist without stent, and LV assist with stent. The threshold of significance was defined as p < 0.05.

## Results

After acute LV failure was established, the novel catheter was smoothly inserted into the LV via the aortic valve in all six goats. No aortic valves in any of the animals showed visible damage at the end of experiments. The rotational speed of the pump was 2200–3300 rpm, which allowed continuous blood flow at 2–3 L/min throughout the experiment. The rate of Ringer’s lactate administration was adjusted according to blood loss during sternotomy in order to provide stable hemodynamics.

### Aortic insufficiency

**Fig 2:**
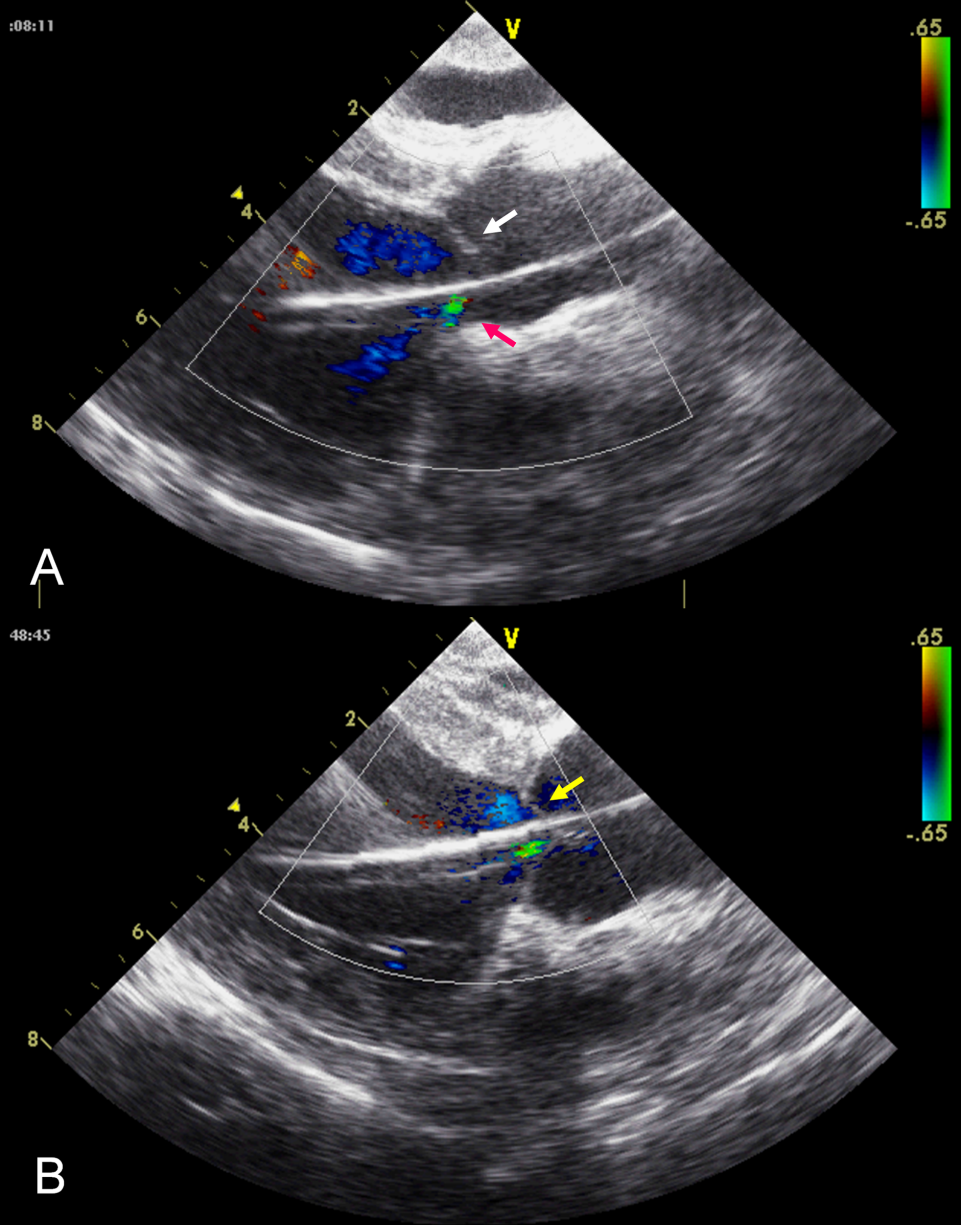
Echocardiograms after insertion of the drainage catheter. **A.** Echocardiogram showing an intact left coronary leaflet (white arrow) and right coronary leaflet compressed by the drainage catheter, generating moderate eccentric aortic insufficiency (red arrow). **B.** Echocardiogram showing that release of the vascular stent keeps the drainage catheter in the center of three valve leaflets. Trivial centric aortic insufficiency was observed in this case (yellow arrow).

AI was not detected in any animal at baseline or after induction of LV dysfunction. Insertion of the catheter into the LV compressed the non-coronary valve of the catheter, which induced mild to moderate AI in all six animals. Initiating the pump aggravated AI until it became a moderate to severe eccentric aortic regurgitation jet toward the anterior mitral leaflet, resulting in aortic cusp distortion (Fig 2A, S2 Video). Release of the retractable stent caused the catheter to move to the central position within the three leaflets of the aortic valve, allowing the leaflets to recover their normal shape and movement. At the same time, eccentric AI disappeared or was mitigated to trivial central AI (Fig 2B, S3 Video). Stent release also prevented the flapping of the drainage catheter caused by the opening and closing of the aortic valve during the cardiac cycle (S4 Video).

### Left ventricle pressure

**Fig 3:**
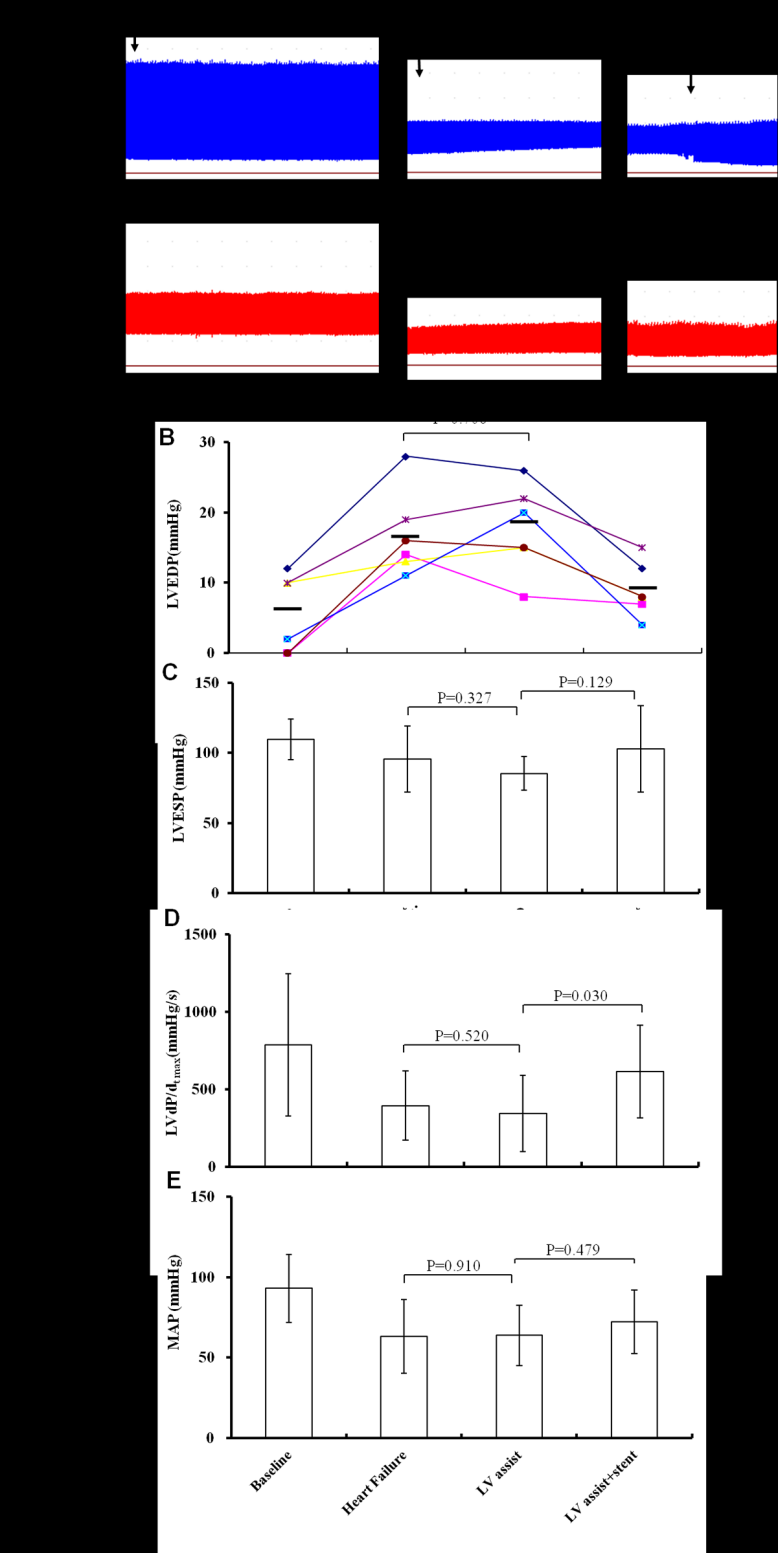
Hemodynamics during LV assist with the novel drainage catheter with and without stent release. **A.** Continuous traces of left ventricle and femoral arterial pressures from one goat. Results shown are representative of those obtained with all six animals. Left ventricle end diastolic pressure (LVEDP) decreased from 26 to 12 mmHg within a few minutes after stent release. **B-E.** Changes in LVEDP, left ventricle end systolic pressure (LVESP), maximum rate of change in left ventricular pressure (d*P*/dt_max_), and mean arterial pressure (MAP). Data are expressed as mean ± SD.

LVEDP increased from 6.8± 4.6 mmHg at baseline to 16.8 ± 4.4 mmHg after induction of acute heart failure (p = 0.011). LVEDP after initiation of LV assist (17.6 ± 5.0 mmHg) was similar to the pressure immediately after heart failure (p = 0.706). After stent release, LVEDP decreased slowly to 9.0±3.0 mmHg within a few minutes, and this value was significantly lower than the corresponding value without stent release (p = 0.012; Fig 3A).

Left ventricle end systolic pressure (LVESP) decreased significantly from 109.5±14.5mmHg at baseline to 95.6±23.5 mmHg after induction of LV failure (p = 0.008). Without stent release, LVESP after initiation of LV assist (85.3±12.1 mmHg) was similar to the value immediately after LV failure (p = 0.327). With stent release, LVESP after LV assist initiation (102.6±30.6 mmHg) was slightly but not significantly higher than the corresponding value without stent release (p = 0.129, Fig 3B). LV assist did not significantly improve mean arterial pressure, regardless of whether the stent was released (Fig 3C).

LV d*P*/d*t*_max_ decreased significantly from 785±460 mmHg/s at baseline to 394±224 mmHg/s after induction of LV failure (Fig 3D). This value did not improve significantly after initiation of LV assist without stent release (343±245 mmHg/s, p = 0.520), but it was significantly higher when the stent was released (614±299 mmHg/s) than when it was not (p = 0.030).

## Discussion

This *in vivo* study presents a novel catheter with a retractable stent for placement in the LV via the aortic valve as part of LV assist. Release of the stent can prop up the catheter against the vessel walls and keep it centered in the aortic valve leaflets, preventing their compression. Such compression can induce aortic insufficiency, which significantly increases LVEDP and compromises systolic function.

Effective LV assist should reduce preload of the LV in order to promote myocardial recovery. This can be accomplished by placing a venting catheter into the LV or left atrium [12]. Some LV assist devices, such as the Impella, are designed to avoid surgical trauma. The Impella, which is widely used in patients with protected percutaneous coronary intervention and acute myocardial infarction, can be inserted percutaneously via the femoral or axillary artery and positioned across the aortic valve into the LV. The Impella and similar devices reduce load on the LV by aspirating blood from the LV into the aorta [13]. A less expensive alternative is a pigtail catheter inserted into the LV via a peripheral artery under echocardiographic or fluoroscopic guidance[14,15], but this may not provide adequate blood flow to decompress the LV without the assistance of extracorporeal membrane oxygenation [16]. In the present study, we provide a potentially more effective and relatively inexpensive alternative in the form of a venting catheter that can easily be inserted into the LV from the left subclavian artery under echocardiographic guidance. When connected to a pump, the catheter provides blood flow at 1.0–1.5 L/min/m^2^ for goats weighing 30–40 kg with estimated body surface areas of 1.02–1.30 m^2^. This blood flow is nearly half that of a typical healthy individual, for whom the cardiac index is 2.82–3.33 L • min^-1^ • m^-2^ [17].

The novel catheter was inserted into the LV and it depressed the aortic valve, triggering AI in all animals, similar to what has been described for the Impella assist [18,19]. We found that this AI offset LV decompression: LV assist in this case failed to reduce LVEDP substantially or improve LVESP or d*P*/dt_max_. This insufficiency also increases risk of leaflet injury, since long-term valve depression by a catheter or pump may lead to permanent aortic insufficiency. This may explain, at least partly, why some patients cannot be weaned off LV support [6,18]. In addition, our catheter pushed against the aortic valve when it moved with the cardiac cycle, as described for the Impella [20]. This can cause valve injury in the case of long-term LV support.

These problems were avoided by releasing a retractable stent at the distal end of the inner tube of our novel catheter. When released, the stent propped up the proximal aorta and fixed the catheter in the center of the valve leaf, eliminating AI and pressure on the valve leaflets, reducing LVEDP and improving LV function. After stent release, the catheter no longer compressed the non-coronary cusp and eliminated catheter movement relative to the valve leaflets. These effects may help reduce the risk of leaflet injury during mid-term LV assist, such as when providing a bridge to recovery or heart transplantation.

This novel catheter is supported by fixing the stent against the wall of the ascending aorta. Therefore, it should be used with caution in patients with diseases involving the ascending aorta or in patients who have undergone coronary artery bypass grafting because of the risk of blocking the opening of the grafted artery. It should also be used cautiously in patients who have severe aortic stenosis or an aortic artificial valve, since proper placement can be difficult.

Despite these potential limitations, the catheter described here is compatible for use with drainage catheters or trans-aortic axial flow pumps. Our results here justify further efforts with larger animal populations to optimize and validate the catheter for clinical use.

## Supporting information

S1 Video Trans-aortic venting of the left ventricle using a novel drainage catheter. A stainless steel guide wire is inserted starting in the peripheral artery, across the aortic valve, and ending in the left ventricle. Under wire guidance, both inner and outer tubes are inserted into the ascending aorta. The inner catheter is inserted into the left ventricle through the aortic valve, then both introducer and guide wire are removed, leaving the outer and inner tubes inside the blood vessel. The outer catheter is withdrawn slightly, releasing the stent.
(AVI)

S2 Video Aortic insufficiency during LV assist without stent. Long-axis view of the left ventricle illustrating aggravation of AI into a moderate to severe eccentric aortic regurgitation jet toward the anterior mitral leaflet. This leads to aortic cusp distortion after initiation of the pump.
(AVI)

S3 Video Aortic insufficiency during LV assist with stent release. Long-axis view of the left ventricle showing that stent release from the drainage catheter eliminates eccentric AI or reduces it to trivial central AI, as well as allows the aortic valve leaflets to recover normal shape and movement.
(AVI)

S4 Video Flapping of the catheter during cardiac cycle. Long-axis view of the left ventricle demonstrating the flapping of the drainage catheter caused by the opening and closing of the aortic valve during cardiac cycle.
(AVI)
